# Metformin Improves Functional Outcomes, Activates Neural Precursor Cells, and Modulates Microglia in a Sex-Dependent Manner After Spinal Cord Injury

**DOI:** 10.1093/stcltm/szad030

**Published:** 2023-05-20

**Authors:** Emily A B Gilbert, Jessica Livingston, Emilio Garcia-Flores, Tarlan Kehtari, Cindi M Morshead

**Affiliations:** Division of Anatomy, Department of Surgery, University of Toronto, Toronto, Canada; Institute of Medical Sciences, University of Toronto, Toronto, Canada; Donnelly Centre for Cellular and Biomolecular Research, University of Toronto, Toronto, ON, Canada; Division of Anatomy, Department of Surgery, University of Toronto, Toronto, Canada; Institute of Medical Sciences, University of Toronto, Toronto, Canada; Donnelly Centre for Cellular and Biomolecular Research, University of Toronto, Toronto, ON, Canada; Division of Anatomy, Department of Surgery, University of Toronto, Toronto, Canada; Institute of Medical Sciences, University of Toronto, Toronto, Canada; Donnelly Centre for Cellular and Biomolecular Research, University of Toronto, Toronto, ON, Canada; Institute of Biomedical Engineering, University of Toronto, Toronto, Canada; Division of Anatomy, Department of Surgery, University of Toronto, Toronto, Canada; Institute of Medical Sciences, University of Toronto, Toronto, Canada; Donnelly Centre for Cellular and Biomolecular Research, University of Toronto, Toronto, ON, Canada; Division of Anatomy, Department of Surgery, University of Toronto, Toronto, Canada; Institute of Medical Sciences, University of Toronto, Toronto, Canada; Donnelly Centre for Cellular and Biomolecular Research, University of Toronto, Toronto, ON, Canada; Institute of Biomedical Engineering, University of Toronto, Toronto, Canada

**Keywords:** spinal cord injury, neural stem cells, oligodendrocyte precursor cells, metformin, microglia, sex characteristics

## Abstract

Spinal cord injury (SCI) results in devastating patient outcomes with few treatment options. A promising approach to improve outcomes following SCI involves the activation of endogenous precursor populations including neural stem and progenitor cells (NSPCs) which are located in the periventricular zone (PVZ), and oligodendrocyte precursor cells (OPCs) found throughout the parenchyma. In the adult spinal cord, resident NSPCs are primarily mitotically quiescent and aneurogenic, while OPCs contribute to ongoing oligodendrogenesis into adulthood. Each of these populations is responsive to SCI, increasing their proliferation and migration to the site of injury; however, their activation is not sufficient to support functional recovery. Previous work has shown that administration of the FDA-approved drug metformin is effective at promoting endogenous brain repair following injury, and this is correlated with enhanced NSPC activation. Here, we ask whether metformin can promote functional recovery and neural repair following SCI in both males and females. Our results reveal that acute, but not delayed metformin administration improves functional outcomes following SCI in both sexes. The functional improvement is concomitant with OPC activation and oligodendrogenesis. Our data also reveal sex-dependent effects of metformin following SCI with increased activation of NSPCs in females and reduced microglia activation in males. Taken together, these findings support metformin as a viable therapeutic strategy following SCI and highlight its pleiotropic effects in the spinal cord.

## Graphical Abstract

**Graphical Abstract:**
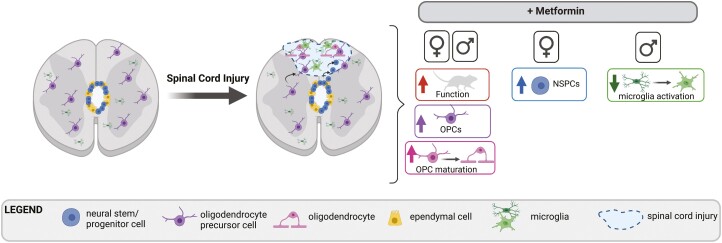

**Significance Statement**

We found that acute metformin treatment leads to behavioral recovery following spinal cord injury (SCI) in both females and males. Metformin elicited sex-dependent effects on microglia and spinal cord stem and progenitor cells with activation of oligodendrocyte precursors and enhanced oligodendrocyte maturation across both sexes. This work identifies metformin as a promising therapeutic to treat SCI in males and females when delivered in the acute phase following injury.

## Introduction

Over 200 000 spinal cord injuries (SCI) occur worldwide each year, and millions of individuals are currently living with the consequences of SCI.^1-3^ Efforts to improve post-SCI recovery include the use of neurotrophic factors, reduction of inhibitory signals, and approaches to enhance neuroplasticity including cell transplantation and cellular reprogramming.^4-10^ To date, recovery remains limited, highlighting the need for novel therapeutic interventions to improve functional outcomes. One potential repair strategy involves the mobilization of resident neural stem and progenitor cells (NSPCs) found in the periventricular zone (PVZ) lining the central canal of the spinal cord, reviewed in ref.^11^ In species capable of spinal cord regeneration (eg, teleost fish, urodeles, and some lizards), resident Sox2+ NSPCs are activated following injury and contribute to the repair of the damaged spinal cord through proliferation, migration, and differentiation to reconstitute the injured or lost tissue.^12-14^ In mammals, SCI also leads to NSPC expansion and migration into the injured parenchyma, but the NSPC response is insufficient to promote functional recovery.^15,16^ Considering their multipotency and responsiveness following injury, harnessing the potential of endogenous NSPCs is a promising approach to promote neural repair and functional recovery in mammals following SCI.^15,17-20^

In addition to NSPCs, oligodendrocyte precursor cells (OPCs) represent a potential target population for enhancing repair following SCI. Chondroitin sulfate proteoglycan (CSPG)- and neural/glial antigen (NG2)-expressing OPCs are found throughout the parenchyma of the spinal cord, comprising 5%-10% of the parenchymal cells and acting as a source of new myelinating oligodendrocytes into ­adulthood.^21-24^ Following SCI, OPCs proliferate and migrate to the site of injury where they contribute to oligodendrogenesis.^25^ However, like the response of NSPCs, the response of OPCs to SCI is insufficient for functional recovery.

Metformin is a safe, FDA-approved drug that is commonly used to treat type II diabetes mellitus.^26^ Metformin has pleiotropic effects in the brain including increased activation of NSPCs and OPCs and reduced microglia activation following injury.^27-31^ Specifically, metformin enhances the proliferation and survival of brain-derived NSPCs in a sex- and age-dependent manner and promotes increased neurogenesis and oligodendrogenesis through its actions on atypical protein kinase C (aPKC)-mediated phosphorylation of CREB-binding protein (CBP).^27,32-35^ Metformin also has anti-inflammatory effects in the CNS when administered following brain injury^30^ including reducing microglia activation following cranial radiation and neonatal hypoxia ischemia.^34,36^ Administration of metformin also leads to improved cognitive and motor outcomes following neonatal stroke.^27,29,35,36^

In the spinal cord, metformin administration can reduce neuropathic pain through activation of (p)-AMPK and suppression of p-STAT3 signaling in the dorsal horn^37^ with effects on microglia activation that were sex-dependent.^38^ In a model of SCI, metformin administration resulted in improved motor outcomes that were associated with increased autophagy^39-42^ and reduced caspase 3 activation.^39^ Following SCI, it is well described that metformin can reduce NF-κB-mediated inflammation, shifting microglia from a pro- to anti-inflammatory state and improving their ability to clear myelin debris.^39,42,43^ Most recently, metformin has also been shown to enhance angiogenesis following SCI, where it drives increased endothelial cell proliferation and higher density of new blood vessels in the spinal cord.^44^ Overall, while metformin’s effects on neural precursor cells have been well described in the brain, metformin’s effects on spinal cord precursor pools have not been clearly established.

Herein, we examined the potential for metformin to promote functional recovery following SCI when administered in both the acute and subacute (delayed) phase post-SCI and examined the neural precursor response that correlated with improved functional outcomes. Given the described sex differences in SCI outcomes^45^ and the sex-dependent effects of metformin in the brain,^29^ we examined all outcomes in both males and females.

## Materials and Methods

### Mice

Mice were group-housed in standard laboratory conditions under a 12:12 h light:dark cycle with ad libitum access to food and water. All procedures were approved by the University of Toronto Animal Care Committee and were performed in accordance with guidelines from the Canadian Council for Animal Care. Male and female cohorts were used and analyzed separately. C57bl6 mice between the ages of 6 and 8 weeks were used (Charles River Laboratories) for in vitro studies. Sox2CreER^T2^ and NG2CreER^T2^ mice crossed with homozygous tdTomato reporter mice (Sox2CreER^T2^tdTomato or NG2CreER^T2^tdTomato) and C57bl6 mice (10-12 weeks of age) were bred in-house and used for in vivo studies.

### Drug Administration

Metformin (1,1-dimethylbiguanide hydrochloride; D150959, Sigma-Aldrich, ON, Canada; 200 mg/kg) was dissolved in sterile phosphate-buffered saline (PBS) and mice received daily intraperitoneal (i.p.) injections for 7 days for all in vitro studies. For in vivo studies, metformin was delivered via subcutaneous (s.c.) mini osmotic pumps (200 mg/kg, 1007D, Alzet, CA, USA) at a rate of 0.5 µL/h for 14 days. Pumps were implanted beginning at the time of SCI (acute) or at 7 days post-SCI (delayed studies). Control mice received sterile PBS via injections (in vitro studies) or mini-pumps (in vivo studies). TdTomato expression was induced 9 days prior to injury via tamoxifen (TAM) chow (250 mg/kg of chow; Envigo, USA).

### Spinal Cord Injury

Mice were anesthetized using isoflurane and given 1 mL lactated ringers, slow-release buprenorphine (1.0 mg/kg), and meloxicam (2.0 mg/kg) prior to SCI.^46,47^ A laminectomy was performed to remove T8/9 vertebrae and expose the spinal cord. Using a surgical microscope, a 25-gauge sterile needle, bent 45^o^ from the shaft, was manually inserted into the cord using the dorsal vein as a landmark. The needle was inserted bilaterally at an oblique angle 0.5 mm lateral to the midline and moved 2 mm parallel to insertion in the rostral direction. The soft tissue overlying the laminectomy site was closed with 6-0 absorbable sutures and the skin was closed with 4-0 silk sutures. Mice were placed on a heating pad and maintained with pain medication (as above) for 48 h. Sham mice were anesthetized using isoflurane and received fluids and pain medication. An incision was made to expose the bony structures and a mini-pump containing sterile PBS was implanted.

### Neurosphere Assay

Mice were anesthetized with isoflurane and euthanized by cervical dislocation. The spinal cord and the PVZ were carefully dissected under a Stemi 2000 microscope (Zeiss, Germany), minced and incubated in papain (Worthington Biochemicals, NJ, USA) dissolved in sterile Earle’s Balanced Salt Solution (EBSS, Worthington Biochemicals). Cells were triturated and spun and then resuspended in a solution containing DNase Ovomucoid protease inhibitor (Worthington Biochemicals), in EBSS and centrifuged. Cells were resuspended in Neurobasal-A media (NBM, Thermo Fisher Scientific, PA, USA) containing l-glutamine (2 mM, Invitrogen, CA, USA), penicillin/streptavidin (100 U/0.1 mg/mL, Invitrogen), epidermal growth factor (EGF, 20 ng/mL; Peprotech, QC, CA); fibroblast growth factor (FGF, 10 ng/mL; Gibco, NY, USA); and heparin (2 µg/mL), Sigma-Aldrich, MI, USA) and centrifuged. Cells were resuspended in culture media in the absence or presence of metformin (0.1, 1, 10, 50, 100, 250, and 500 ng/mL). Cells were plated at a density of 10 cells/µL in 24-well plates (Thermo Fisher Scientific) in media for 7 days at 37 C and 5% CO_2_. The numbers of neurospheres (>80 µm) were counted in 6 wells of a 6 well plate (Sigma-Aldrich) and averaged per animal.

### Neurosphere Differentiation

Individual neurospheres were collected and plated into laminin-coated wells in a 48 well plate (Sigma-Aldrich). Each sphere was plated in NBM containing 1% fetal bovine serum (FBS; Wisent Bioproducts, QC, Canada) and 100 ng/mL metformin for 7 days at 37 °C and 5% CO_2,_ then fixed using ice-cold 4% paraformaldehyde (PFA) for 20 min, rinsed in PBS and stored at 4 °C until processed for immunohistochemistry.

### In Vitro Immunohistochemistry

Cells were washed in PBS and then blocked for 1 h in 10% blocking solution (normal goat serum (NGS) in PBS). Cells were incubated in primary antibody O4 (oligodendrocytes; 1:1000; R&D Systems, MAB1326; see Table 1) in blocking solution, overnight at 4 °C. The following day cells were washed in PBS and incubated in secondary antibody (goat anti-mouse 568 IgM, 1:400; Thermo Fisher Scientific, A21043) in blocking solution, for 1 h followed by PBS washes. Cells were permeabilized using 0.3% Triton-X-100 in PBS for 20 min prior to PBS washes and exposed to blocking solution for 1 at room temperature then incubated in primary antibodies TUJ1 (for neurons; 1:1000; Sigma-Aldrich, T8660), GFAP (for astrocytes; 1:500; Sigma-Aldrich, G9269); see also (Table 1) in blocking solution overnight at 4 ^°^C. The next day, cells were washed in PBS and incubated with secondary antibodies (goat anti-mouse 488 IgG, 1:400; Thermo Fisher Scientific, A11034 and goat anti-rabbit 647 IgG; 1:400; Thermo Fisher Scientific, A21247). Following PBS washes the nuclear stain DAPI (1:10000; Thermo Fisher Scientific, D1306) was added for 5 min then cells were rinsed in PBS.

**Table 1.** Antibody information.

Antigen	Supplier	Cat#	Host	Dilution	RRID
O4S	R&D systems	MAB1326	Mouse	1:1000	AB_357617
TUJ1	Sigma-Aldrich	T8660	Mouse	1:1000	AB_477590
GFAP	Sigma-Aldrich	G9269	Rabbit	1:500	AB_477035
IBA-1	Wako	019-19741	Rabbit	1:500	AB_839504
APC	EMD Millipore	OP80	Mouse	1:50	AB_2057371

Imaging was performed using a Zeiss microscope (Axiovert 200 M). To quantify the relative proportions of neurons, oligodendrocytes, and astrocytes from 4 separate images were counted from 5 individually plated neurospheres across each biological replicate. Percentages of differentiated cells were calculated as a proportion of the total DAPI+ cells within each image.

### EdU Administration

5-Ethynyl-2ʹ-deoxyuridine ((EdU); E10187, Fisher Scientific, Pittsburgh, PA, USA) was administered (50 mg/kg) via i.p. injections once daily on days 4-6 following SCI.

### Tissue Processing and Immunohistochemistry

At the time of euthanasia, mice were injected with an overdose of Avertin (Sigma-Aldrich) and transcardially perfused with ice-cold PBS, followed by 4% PFA. Spinal cords were removed and post-fixed in 4% PFA and then transferred to 30% sucrose in PBS. Tissue was embedded with OCT compound (Thermo Fisher Scientific, MA, USA) and frozen. Spinal cords were cryosectioned (10 μm), collected onto SuperfrostPlus slides (Thermo Fisher Scientific), and stored at −20 °C until use.

Slides were rinsed in PBS and then treated in 0.3% Triton X-100 in PBS and then rinsed in PBS. Slides were blocked in 0.1% bovine serum albumin and 5% NGS in 0.3% Triton X-100 PBS. Slides were then incubated in primary antibodies as shown in Table 1 overnight at 4 °C. The following day, slides were rinsed in PBS and incubated with secondary antibody (goat anti-mouse 488 IgG; 1:400 Thermo Fisher Scientific, A11001 or goat anti-rabbit 488 IgG; 1:400 Thermo Fisher Scientific, A11034), rinsed in PBS and then incubated in nuclear stain (DAPI, 1:10 000; Thermo Fisher Scientific, D1306). Slides were rinsed again in PBS and cover slipped using fluorescent mounting media (Agilent, S302380-2).

To detect Edu+ cells, the Click-it kit was used (Thermo Fisher Scientific, C10634) following the manufacturer’s instructions. Slides were placed in 0.3% Triton X-100 (Sigma-Aldrich, ON, Canada) for 20 min and incubated with the Click-It reaction cocktail for 30 min which was composed of Tris-buffered saline (6.05 g Tris (17 926, Thermo Fisher Scientific) + 8.76 g NaCl (SOD001.1, BioShop, Canada) + 800 mL ddH_2_O; pH of 7.5), Copper Sulfate (2411A, Sigma-Aldrich), 647 Azide (A10277, Thermo Fisher Scientific) and 1X reaction buffer (C10634, Thermo Fisher Scientific). Slides were rinsed in PBS and incubated in nuclear stain (DAPI,1:10 000; Thermo Fisher Scientific, D1306). Slides were rinsed again in PBS and cover slipped using fluorescent mounting media (Agilent, S302380-2).

Imaging was performed using Zeiss microscopes (Axiovert 200 M or spinning disc confocal; Zeiss, Germany) or an EVOS M5000 imaging system (Thermo Fisher Scientific, USA). Immuno-positive cells were counted from 6 to 8 transverse sections per mouse bilaterally through the lesion site in the dorsal columns or the PVZ. One section per 50 μm from the core of the lesion outwards, both rostrally and caudally, was quantified. Across all cellular analyses, the region of interest (ROI) was 450 μm^2^ (unit area). For each of the 6-8 sections/animal, we averaged 12-16 ROI’s to get one value per spinal cord/mouse.

### Functional Assessments

Functional assessments took place in a dedicated behavioral testing room. Mice were acclimated to the behavioral suite for at least 30 min before testing. Behavior was analyzed by an experimenter blinded to group assignment (Supplementary Table S1). Baseline performance was established and mice were tested weekly following SCI.

#### Raised Horizontal Ladder

The horizontal ladder test was used to evaluate skilled coordination. Mice were acclimated in a goal box for 2 min and then crossed unevenly spaced rungs on a raised ladder for 3 consecutive trials. The number of hind limb steps and slips were counted and the percentage of slips was calculated.

#### Gait Analysis

Gait analysis was performed prior to using the Noldus Catwalk-XT (Noldus, The Netherlands). Mice were acclimated in the goal box for 2 min and then prompted to cross a glass walkway for 3 consecutive trials. A maximum speed variation of 60% was allowed for each run. Various gait parameters were analyzed using Noldus Catwalk-XT software. The fold change in each parameter of interest was calculated by normalizing experimental data to the average of controls.

### Statistical Analyses and Exclusions

All statistical analyses were conducted using SPSS (v. 23; IBM) and Prism (v. 9; GraphPad) software. Neurosphere data from dose–response curves were transformed into fold change compared to the average control (vehicle treated) value and analyzed using a one-way analysis of variance (ANOVA) followed by Bonferroni post hoc tests. Neurosphere data from in vivo metformin studies was transformed into fold change compared to the average Sham value and analyzed using a 2-way ANOVA followed by Šidák’s multiple comparison test. In vitro cellular differentiation data were analyzed using unpaired *t*-tests. Catwalk data were transformed into fold change compared to Sham controls, and Catwalk and horizontal ladder data were analyzed using repeated-measures ANOVA followed by Šidák’s or Dunnett’s post hoc tests comparing back to baseline performance. All in vivo immunohistochemical data were analyzed using a one-way ANOVA followed by Tukey’s post hoc tests. A *P*-value of <.05 was considered significant. For cellular analyses, a subset of animals from each group was sampled. Mice that did not display functional impairments following SCI were excluded from the behavioral analyses (*n* = 4 mice in the delayed metformin studies that showed no deficit on PID7 before drug or vehicle treatment).

## Results

### Acute Metformin Treatment Improves Outcomes Following SCI

**Figure 1.**
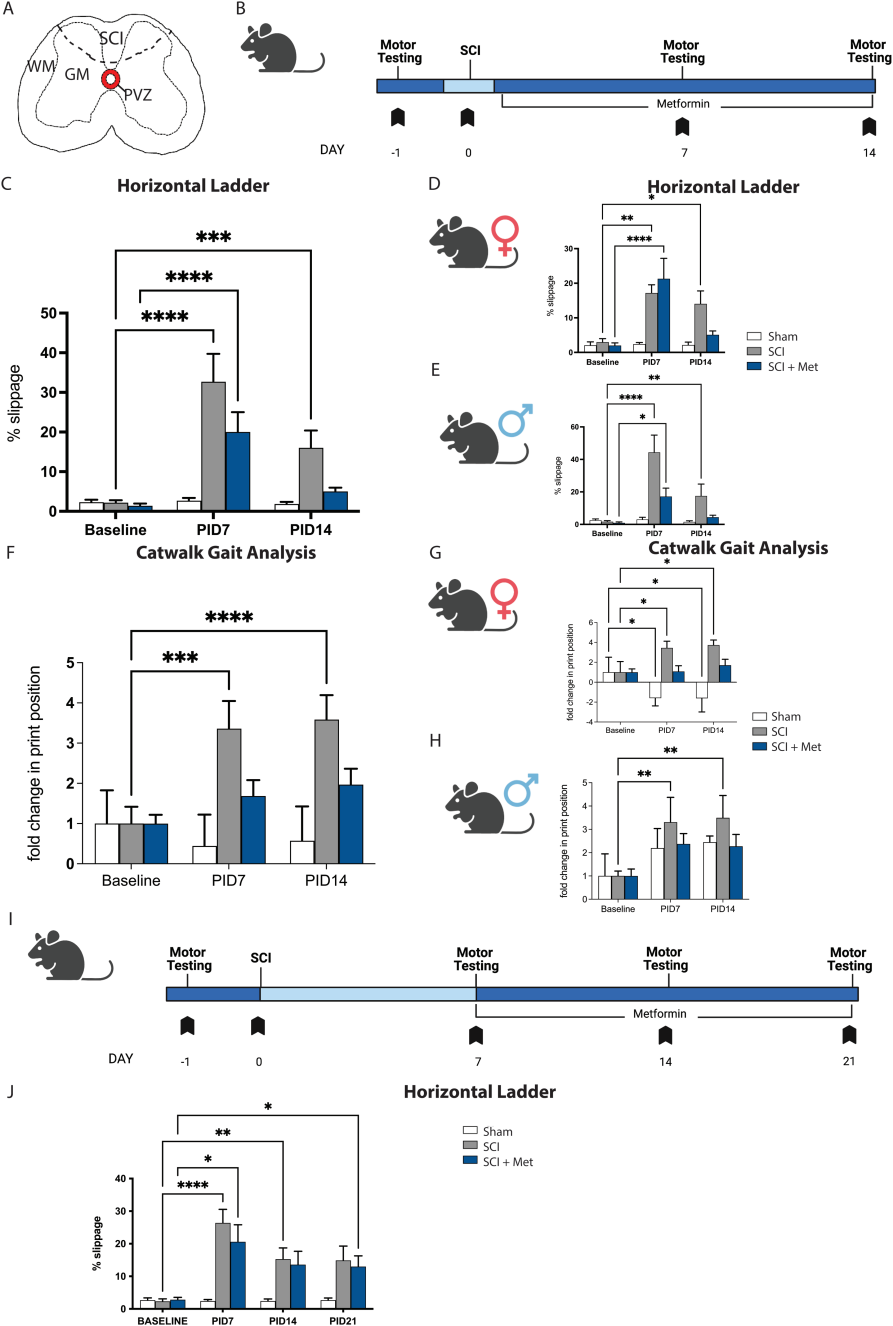
Immediate metformin improves functional recovery following SCI (**A**) Transverse schematic of the spinal cord, hatched line indicates the site of SCI. (**B**) Experimental timeline for immediate metformin study. **(C)** SCI mice are impaired in the horizontal ladder task at PID7 and PID14 (increased % slippage). SCI + Met mice are significantly impaired on PID7 and by PID14 they have recovered to Sham controls. **(D)** In females, SCI and SCI + Met treated mice are impaired on PID7. SCI + Met treated females recovered by PID14 and are not significantly impaired compared to Sham mice. **(E)** Male mice display a significant deficit on PID7 following SCI and SCI + Met. SCI + Met treated males recover by PID14 and are not significantly impaired compared to Sham mice. **(F)** Gait analysis revealed significant changes in hind paw print width at PID7 and PID14 in SCI mice. SCI + Met mice were not significantly impaired at either PID7 or PID14. **(G**) SCI females demonstrated impaired gait at PID7 that persisted to PID14. SCI + Met treated females were not significantly impaired at PID7 or PID14. **(H)** A significant deficit is observed in SCI males on PID7 and PID14. SCI + Met treated males were not significantly impaired at PID7 or PID14. **(I)** Experimental timeline for delayed (subacute) metformin study. **(J)** Mice were significantly impaired (increased % slippage) following SCI, with or without metformin treatment, on PID7, PID14, and PID21. WM, white matter, GM, gray matter, PVZ, periventricular zone, SCI, spinal cord injury. Data are presented as mean ± SEM; combined data, *n* = 14-25/treatment group; separated by sex, *n* = 6-13 males and females/treatment group. *P < .05, **P < .01, ***P < .001, ****P < .0001.

We first sought to determine whether metformin treatment could improve functional outcomes following SCI. To perform these studies, we utilized a previously described minimally invasive model of SCI (Fig. 1A).^18^ Mice received a SCI and were implanted with subcutaneous pumps to deliver metformin for 14 days beginning at the time of SCI (“acute”) (Fig. 1B). Functional assessment was examined using 2 motor tasks: the raised horizontal ladder and the Noldus Catwalk-XT system. Mice were tested on both tasks prior to injury (baseline) and on post-injury day (PID) 7 and PID 14 (Fig. 1B). Given the reported sex-dependent responses to SCI^48,49^ and sex-dependent responses to drug treatments in the CNS, including metformin,^29,38^ we analyzed the data as combined (pooled sexes) and in males and females separately.

When combined, SCI-injured mice displayed a significant deficit compared to baseline on the horizontal ladder test at PID7 (baseline = 2.21 ± 0.59% slips, PID7 = 32.70 ± 7.05% slips, *P* < .0001) which persisted until PID14 (16.03 ± 4.36% slips, *P* = .0006). Mice that received metformin treatment starting at the time of injury were also significantly impaired at PID7 (baseline = 1.41 ± 0.56% slips, PID7 = 20.04 ± 4.99% slips, *P* < .0001) (Fig. 1C), but recovered to baseline performance by PID14 (5.01 ± 0.98% slips, *P* = .5341) (Fig. 1C).

When we analyzed sexes separately, female mice were impaired on the ladder test at PID7 regardless of metformin treatment (SCI baseline = 2.97 ± 1.01% slips, SCI PID7 = 17.19 ± 2.61% slips, *P* = .009; SCI + Met baseline = 1.99 ± 0.78% slips; SCI + met PID7 = 22.0 ± 8.24% slips, *P < .*001) (Fig. 1D). At PID14, mice that received metformin were recovered while SCI mice remained impaired (SCI = 14.05 ± 4.10% slips, *P* = .0499; SCI + Met = 5.40 ± 1.53% slips, *P* = .578) (Fig. 1D). A similar finding was seen in males whereby mice were impaired following SCI at both PID7 (baseline = 1.63 ± 0.70% slips, PID7 = 44.33 ± 10.64% slips; *P* < .0001) and PID14 (SCI = 17.52 ± 7.32% slips, *P* = .007) (Fig. 1E). Males that received metformin treatment were significantly impaired on PID7 (21.78 ± 5.13% slips, *P* = .017) but recovered by PID14 (11.60 ± 7.28% slips, *P* = .803) (Fig. 1E). Hence, metformin rescued the functional deficits that resulted following SCI in both males and females.

Functional outcomes were further investigated by gait analysis using the Catwalk. Gait measures of SCI-injured mice were normalized to baseline sham performance to detect any differences at PID7 and PID14 compared to their pre-injury performance (Fig. 1B). In the untreated SCI group, there was a significant deficit in hind paw print position detected at PID7 (3.36 ± 0.69 fold change, *P* = .0003) and PID14 (3.59 ± 0.61 fold change; *P* < .0001). Following metformin treatment, this deficit was abrogated at both time points (PID7, 1.69 ± 0.40 fold change, *P = .*466; PID14, 1.98 ± 0.39 fold change, *P* = .224) (Fig 1F).

Sex analysis revealed impaired gait in females at PID7 (3.43 ± 0.68 fold change; *P* = .038) and the impairment persisted at PID14 (3.73 ± 0.51 fold change, *P* = .019). Following metformin treatment, no impairment was observed at either time (PID7, 1.08 ± 0.58 fold change, *P = .*995; PID14, 1.70 ± 0.60 fold change, *P = .*676) (Fig. 1G). Sham animals showed a change in print position over time, however, this was in the form of a reduction from baseline (PID7 = −1.560 ± 0.78 fold change, *P* = .042; PID14 = −1.61 ± 1.38 fold change, *P* = .040).

Males displayed a significant deficit in gait following SCI at PID7 (3.31 ± 1.07 fold change, *P* = .004) and PID14 (3.49 ± 0.96 fold change, *P* = .002) and this impairment was abrogated following metformin treatment (PID7, 2.37 ± 0.45 fold change, *P* = .187; PID14, 2.27 ± 0.51 fold change, *P* = .233) (Fig. 1H). A number of other gait impairments were improved following metformin treatment in one or both sexes, including phase dispersion and couplings (measurements of the relationships between the paws during step cycles), and hind stride length^50^ (Supplementary Fig. S1), further supporting the functional benefits of post-injury metformin treatment.

### Delayed Metformin to the Subacute Phase Does Not Improve Functional Outcomes Following SCI

We next asked if metformin could improve functional outcomes when administration was delayed following injury. In this paradigm, metformin treatment began at PID7 and was continued for 14 days, until PID21 (Fig. 1I). SCI mice show a significant deficit at PID 7 (26.39 ± 4.16% slips; *P* < .0001 compared to baseline; 2.32 ± 3.05% slips) that persisted at PID14 (15.28 ± 16.63% slips; *P* = .0041) and PID21 (14.88 ± 17.65% slips; *P* = .031). SCI + Met groups also showed a significant deficit compared to PID7 (20.59 ± 5.22% slips; *P* = .012). This persisted through PID14 (13.58 ± 4.09% slips, *P* = .049) and PID21 (12.99 ± 3.28% slips, *P* = .019) (Fig. 1J). Hence, we observed no improvement in functional outcomes in SCI + Met treated mice when metformin administration was delayed to the subacute phase post-SCI. Given the efficacy of the acute metformin treatment paradigm in improving motor outcomes, we next explored the cellular effects at the time of functional recovery.

### Metformin Administration Expands NSPCs in the Spinal Cord

To determine whether NSPCs in the adult spinal cord were responsive to metformin we performed the in vitro colony forming neurosphere assay from separate cohorts of female and male mice.^51-53^ Primary cells from the NSPC niche (the PVZ) were plated in the presence or absence of metformin (ranging from 0 to 500 ng/mL) for 7 days and the number of neurospheres was quantified (Supplementary Fig. S2A). Across males and females, we did not observe differences in the total number of neurospheres in untreated controls (females = 19.17 ± 5.34 spheres, males = 24.08 ± 5.94 spheres, *P* = .999). We observed a significant increase in the numbers of female-derived neurospheres at 100 ng/ml and 500 ng/mL of metformin relative to controls (3.61 ± 0.40 fold increase at 100 ng/ml, *P* = .0004; 3.33 ± 1.33 fold increase at 500 ng/ml, *P* = .017) and a significant increase in the number of neurospheres in males at 100 ng/mL (2.86 ± 0.51 fold increase, *P* = .024) (Supplementary Fig. S2B, S2C). Hence, NSPCs derived from the spinal cord of males and females are responsive to metformin administration in vitro.

**Figure 2.**
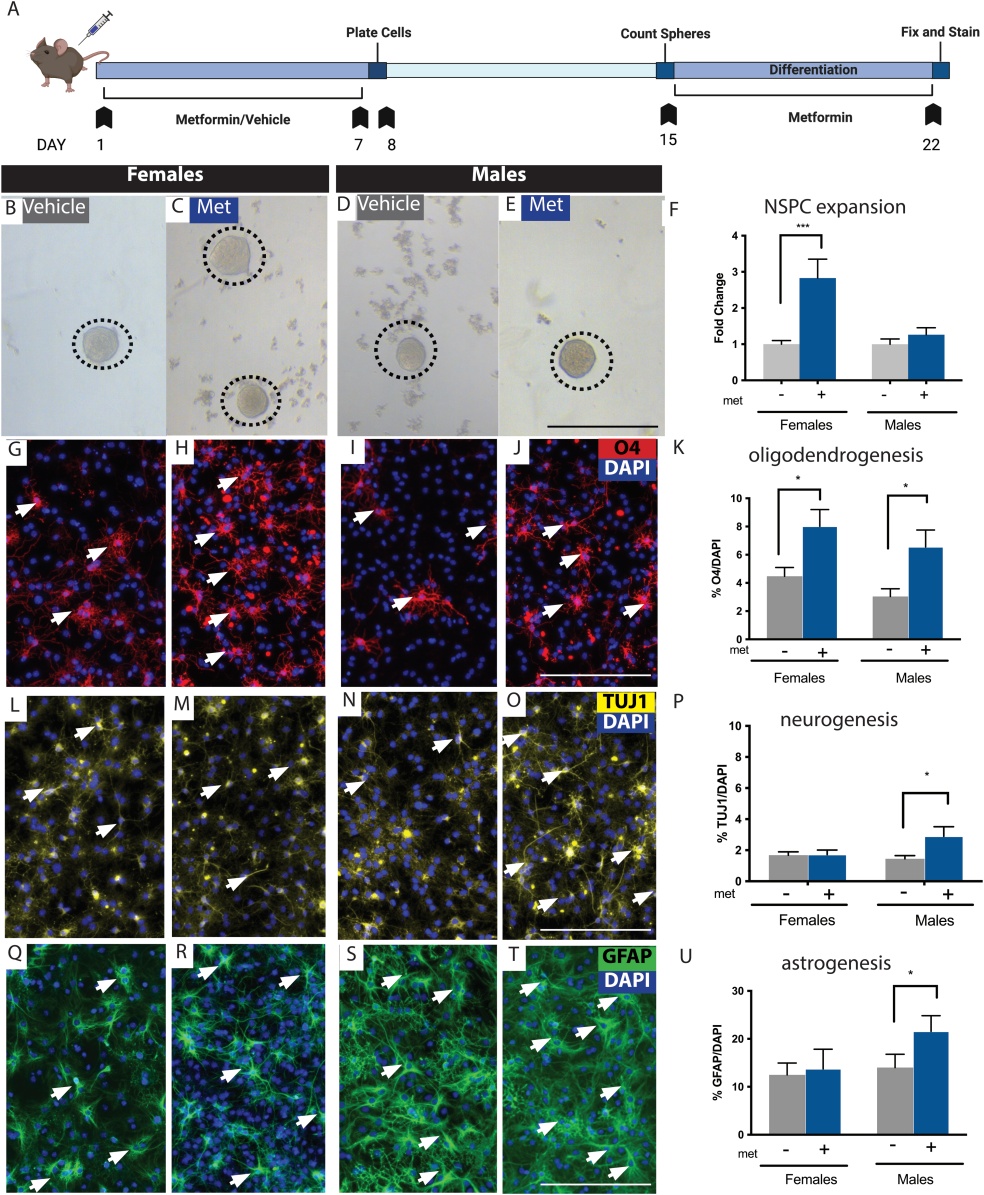
Metformin expands the NSPC pool and promotes oligodendrogenesis and neurogenesis *in vitro.***(A)** Experimental timeline. **(B-E)** Spinal cord-derived neurospheres are present from vehicle- and metformin-treated mice. **(F)** Females (but not males) have significantly more neurospheres following metformin treatment compared to vehicle treatment, **(G-J)** Immunohistochemistry for the oligodendrocyte marker 4 (O4). **(K)** Quantification reveals a significantly greater percentage of O4+ oligodendrocytes (arrows) in both males and females with metformin treatment. **(L-O)** Immunohistochemistry for neurons using ß-III-Tubulin (TUJ1). **(P)** We observed significantly higher percentages of neurosphere derived TUJ1 neurons (arrows) from metformin-treated males, but not females. **(Q-T)** Immunohistochemistry for astrocytes using GFAP. **(U)** We observed no change in astrogliogenesis in females, but significantly more GFAP+ astrocytes in males following metformin administration (arrows). Scale bar in E, J, O, T = 200 μm. Data are presented as mean ± SEM; *n* = 12 neurospheres/treatment group from 9 mice/group/sex. *P < .05.

We next administered metformin to naïve adult female and male mice for 7 days via osmotic minipump to ask whether NSPCs were expanded following in vivo drug exposure (Fig. 2A). The neurosphere assay was performed 1 day following treatment. Interestingly, metformin administration resulted in an increase in the number of neurospheres from females (2.83 ± 0.53 fold increase, *P* = .0001, Fig. 2B, 2C, 2F), but not males (1.26 ± 0.19 fold increase; *P* = .7789; Fig. 2D–2F), compared to saline-treated controls. Thus, in vivo administration of metformin results in expansion of the spinal cord NSPC pool in a sex-dependent manner.

### Metformin Administration Alters the Differentiation Profile of NSPCs

To further explore the effects of metformin on spinal cord-derived NSPCs, we characterized the differentiation profile of single neurospheres derived from metformin or vehicle-treated mice. Neurospheres were grown in the absence of metformin and subsequently differentiated for 7 days in the presence of metformin (100 ng/mL) (Fig. 2A). The phenotype of differentiated cells was assessed using immunohistochemistry for TUJ1 (neurons), O4 (oligodendrocytes), and GFAP (astrocytes). All neurospheres were multipotent (Supplementary Fig. S3A, S3D). The percentage of oligodendrocytes was significantly greater in metformin-treated females (vehicle = 4.47 ± 0.63% O4+ cells, Met = 8.01% ± 1.20% O4+ cells, *P* = .0128) and males (vehicle = 3.03 ± 0.55% O4+ cells, Met = 6.55 ± 1.20% O4+ cells, *P* = .0068) derived neurospheres (Fig. 2G–2K). Metformin treatment did not affect neurogenesis from female-derived neurospheres (vehicle = 1.68 ± 0.21% TUJ1+ cells, Met = 1.72 ± 0.29% TUJI+ cells, *P* = .9213), while male-derived neurospheres had increased numbers of TUJI+ neurons (vehicle = 1.45 ± 0.20% TUJI+ cells, Met = 2.89 ± 0.62% TUJI+ cells, *P* = .0193) (Fig. 2L–2P). We also observed a sex-dependent increase in the percentage of GFAP+ astrocytes following metformin treatment in males (vehicle = 14.02 ± 2.76% GFAP+ cells, Met = 21.55 ± 3.29% GFAP+ cells, *P* = .0003) but not females (vehicle = 12.48 ± 2.78% GFAP+ cells, Met = 13.73 ± 4.10% GFAP+ cells, *P* = .8834) (Fig. 2Q–2U) These findings are consistent with metformin’s ability to modulate the differentiation profile of brain-derived NSPCs in vitro^27,32,33^ and further reveal sex-dependent effects of metformin on spinal cord derived NSPC differentiation.

### Metformin Enhances the Number of NSPC-Derived Cells in the Injured Parenchyma

**Figure 3.**
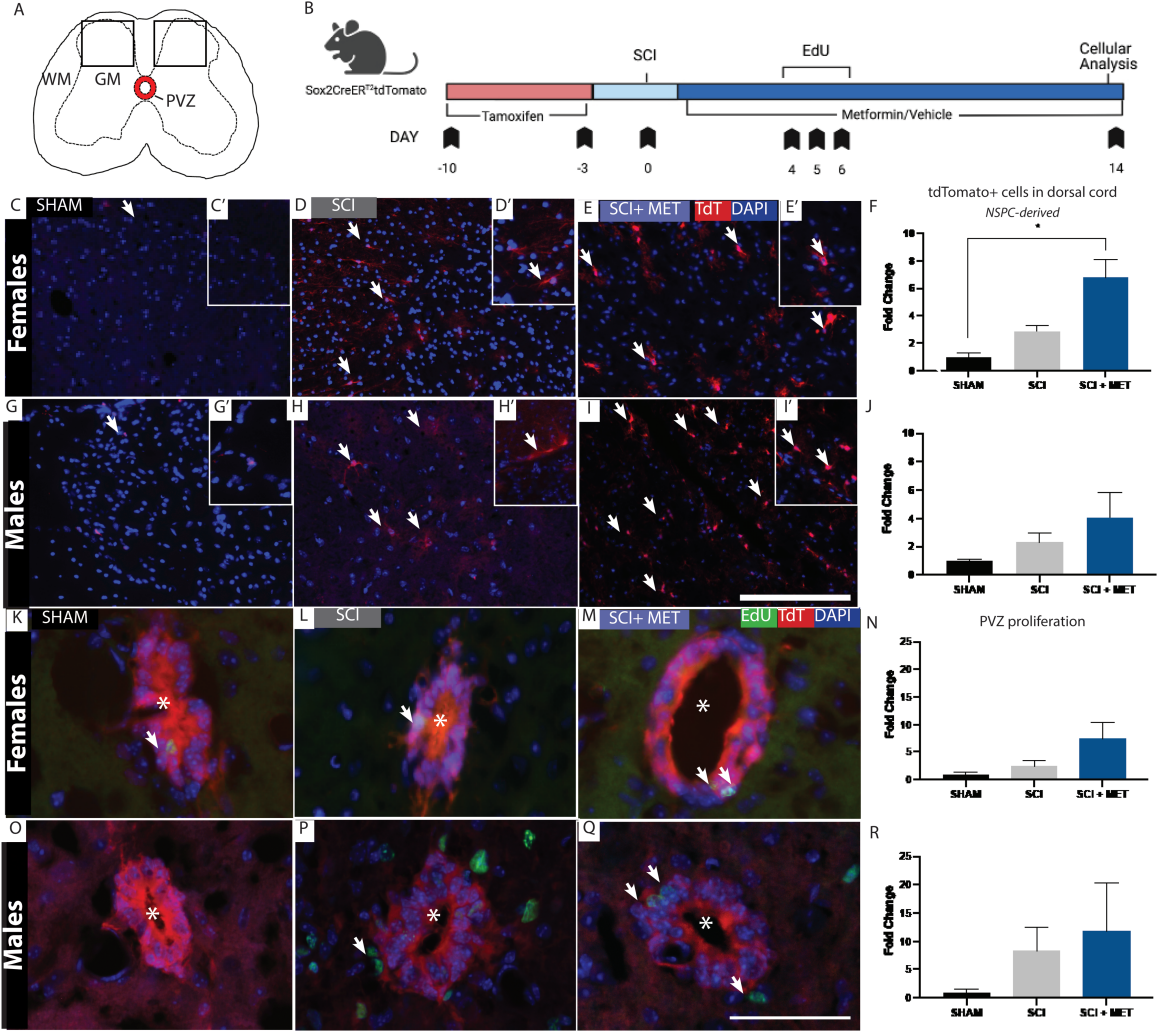
Metformin enhances the number of NSPC-derived cells in the injured parenchyma of female mice at the time of functional recovery. (**A**) Transverse schematic of the spinal cord. Sox2+ NSPCs were pre-labeled in the PVZ (outline) and tracked following SCI. Boxes represent the regions of interest counted in each of the sections (450 μm^2^). **(B)** Experimental timeline. **(C-E)** Lineage tracking of TdTomato+, Sox2-derived progeny in Sham (**C-C**ʹ**),** SCI **(D-D**ʹ**),** and SCI + Met **(E-E**ʹ**)** in females. Hatched line outlines the PVZ. Arrows indicate PVZ-derived tdTomato+ cells in the parenchyma at PID14. **(F)** Significantly more tdTomato+ cells are in the parenchyma of SCI + Met treated females compared to Sham controls. **(G-I)** Lineage tracking of tdTomato+, Sox2-derived progeny in Sham (**G-G**ʹ**),** SCI **(H**-**H**ʹ****), and SCI + Met **(I-I**ʹ**)** treated males. **(J)** Males did not have a significant increase in tdTomato+ NSPC derived cells in the parenchyma following SCI or SCI + Met. **(K-M)** EdU+ NSPCs (arrows) in the PVZ (*indicates central canal) were assessed in Sham **(K),** SCI **(L),** and SCI + Met **(M)** treated females. **(N)** There is no significant increase in EdU+ cells in the PVZ in females. **(O-Q)** EdU+ cells in the PVZ of Sham **(O)**, SCI **(P)** and SCI + Met treated males. **(R)** There was no significant increase in EdU+ cells in males across treatment groups. Scale bar in I = 250 μm, scale bar in Q = 50 μm. Data are presented as mean ± SEM; *n* = 3-6 mice/group. *P < .05.

To examine the cellular effects of metformin administration following SCI and recovery, we used an inducible Sox2-CreER^T2^tdTomato transgenic mouse line to label and track NSPCs from the PVZ in female and male mice by visualizing the presence of tdTomato expressing cells (Fig. 3A). Tissue analysis was performed on PID14 (Fig. 3B). We quantified the numbers of tdTomato+ cells in the dorsal spinal cord of Sham, SCI, and SCI + Met treated groups. As predicted, the vast majority of tdTomato+ cells were confined to the PVZ in Sham mice of both sexes (Fig. 3C, 3F, 3G, 3J). Following SCI, tdTomato+ cells were observed in the dorsal parenchyma in both SCI and SCI+Met groups (Fig. 3D–3J). Quantification in females revealed a significant increase in tdTomato+ cells in the dorsal parenchyma of SCI + Met treated mice compared to Sham controls (Sham = 1.00 ± 0.25 vs. SCI + Met = 6.79 ± 1.30 fold change, *P* = .0114) (Fig. 3F). There was also a trend toward higher numbers of tdTomato+ cells in the injured parenchyma of males in SCI + Met treated group compared to Sham controls (Sham = 1.00 ± 0.08 vs. SCI + Met = 4.09 ± 1.76 fold change, *P* = .225) (Fig. 3J). Notably, very few of the tdTomato+ PVZ-derived cells in the dorsal parenchyma were proliferating at PID14, irrespective of sex, and there was no difference in EdU+/tdTomato+ cells in SCI vs. SCI + Met groups (Supplementary Fig. S4). Hence, metformin treatment enhances NSPC activation in the PVZ following SCI.

To assess whether metformin administration increased the proliferation of NSPCs in the PVZ (Fig. 3A), male and female Sox2-CreER^T2^tdTomato mice received daily EdU injection at early times post-SCI (PID4-6) (Fig. 3B). The numbers of EdU+/TdTomato+ cells in the PVZ was quantified across Sham, SCI, and SCI + Met groups on PID14. In both females and males, there was a trend toward increased EdU+ cells in the PVZ but no significant increase was detected in either sex (Fig. 3K–3R).

### Metformin Enhances the Numbers of OPC-Derived Cells and Drives OPC Maturation in the Injured Parenchyma Following SCI

**Figure 4.**
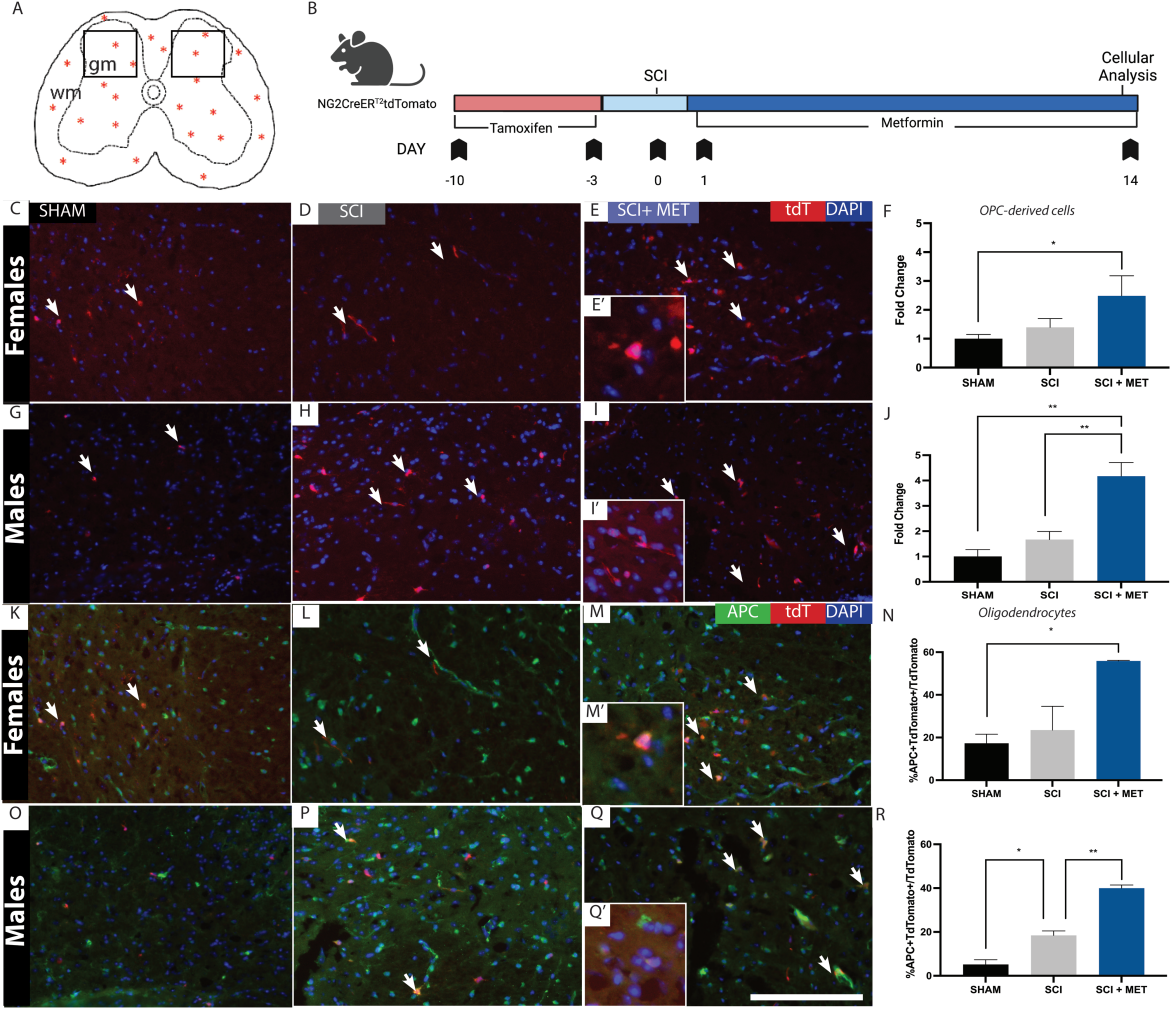
Metformin increases the number of OPC-derived cells and enhances OPC maturation. (**A**) Transverse schematic of the spinal cord. NG2+ OPCs dispersed within the parenchyma (stars) were pre-labeled in NG2CreERT2:TdTomato mice and tracked at PID14. Boxes represent the regions of interest counted in each of the sections (450 μm^2^)**. (B)** Experimental timeline. **(C-E)** tdTtomato+ OPC-derived progeny (arrows) in female Sham **(C)**, SCI **(D)**, and SCI + Met **(E-E**ʹ**)** mice. **(F)** There is a significant increase in the number of tdTomato+ cells in the injured parenchyma in SCI+Met treated female mice compared to Sham. **(G-I)** tdtomato+ OPC-derived progeny (arrows) in the parenchyma of Sham **(G)**, SCI **(H)**, and SCI + Met **(I-I’)** male mice. **(J)** There is a significant increase in the number of tdTomato+ cells in the injured parenchyma in SCI + Met mice compared to both Sham and SCI. **(K-M)** tdTomato+APC+ cells (arrows) were identified across Sham **(K)**, SCI **(L),** and SCI + Met **(M-M**ʹ**)** female mice. **(N)** A significant increase in the percentage of tdTomato+APC+/tdTomato+ cells was observed in SCI+Met-treated females. **(O-Q)** tdTomato+/APC+ cells (arrows) were identified across Sham **(O)**, SCI **(P)** and SCI + Met **(Q-Q**ʹ**)** treated male mice. **(R)** The percentage of tdTomato+APC+/tdTomato+ cells was increased following SCI and in SCI+Met treated males. Scale bar in Q = 150 μm. Data are presented as mean ± SEM; *n* = 3-6 mice/group. *P < .05.

We next asked whether metformin treatment increased the number of OPC-derived cells in the injured parenchyma following SCI. We used NG2-CreER^T2^tdTomato transgenic mice to label and track OPCs in both female and male mice (Fig. 4A, 4B). We quantified the numbers of tdTomato+ cells in the dorsal spinal cord of Sham, SCI, and SCI + Met treated mice on PID14 (Fig. 4C, 4E, 4G, 4I). In females, we observed a significant increase in the number of OPC-derived cells in the injured parenchyma in SCI + Met mice (Sham = 1.00 ± 0.15 vs. SCI + Met = 2.49 ± 0.70 fold increase, *P* = .0433) (Fig. 4F). In males, we also observed a significant increase in the number of OPC-derived cells in the injured parenchyma in SCI + Met treated mice relative to both sham (*P* = .0012) and SCI (*P* = .0068) groups (Sham = 1.00 ± 0.27 vs. SCI = 1.67 ± 0.32 vs. SCI + Met = 4.17 ± 0.54 fold increase) (Fig. 4J). Hence, metformin expands the OPC pool following SCI in both sexes.

To determine whether metformin promoted the differentiation of NG2+ OPCs into mature oligodendrocytes, we quantified the number of mature oligodendrocytes (APC+) originating from tdTomato+ OPCs (APC+/tdTomato+) cells in the injured parenchyma (Fig. 4K–4R) in the presence or absence of metformin treatment. In females, a significant increase in the percentage of APC+/tdTomato+ cells in SCI + Met mice was observed compared to Sham controls (Sham = 17.29 ± 4.24% APC/tdTomato cells; SCI + Met = 55.90 ± 0.27% APC+/tdTomato+ cells, *P* = .024) (Fig. 4N). In males, we observed a significant increase in the percentage of APC+/TdTomato+ cells (Fig. 4O–4R) in SCI mice compared to Sham (Sham = 5.20 ± 2.14% APC+/TdTomato+ cells, SCI = 18.43 ± 2.06% APC+/TdTomato+ cells, *P* = .0211) and a significant increase in the percentage of APC+/TdTomato+ cells in SCI + Met mice compared to both Sham and untreated SCI mice (SCI + Met = 39.96 ± 1.50% APC+/tdTomato+ cells, *P* = .0008 (compared to sham), *P* = .0037 (compared to SCI)). These findings reveal that spinal cord OPCs in both sexes are responsive to metformin treatment following SCI, expanding in number and generating mature oligodendrocytes.

### Metformin Does Not Alter the Number of Microglia Following SCI, But Reduces Ameboid Microglia in Males Following SCI

**Figure 5.**
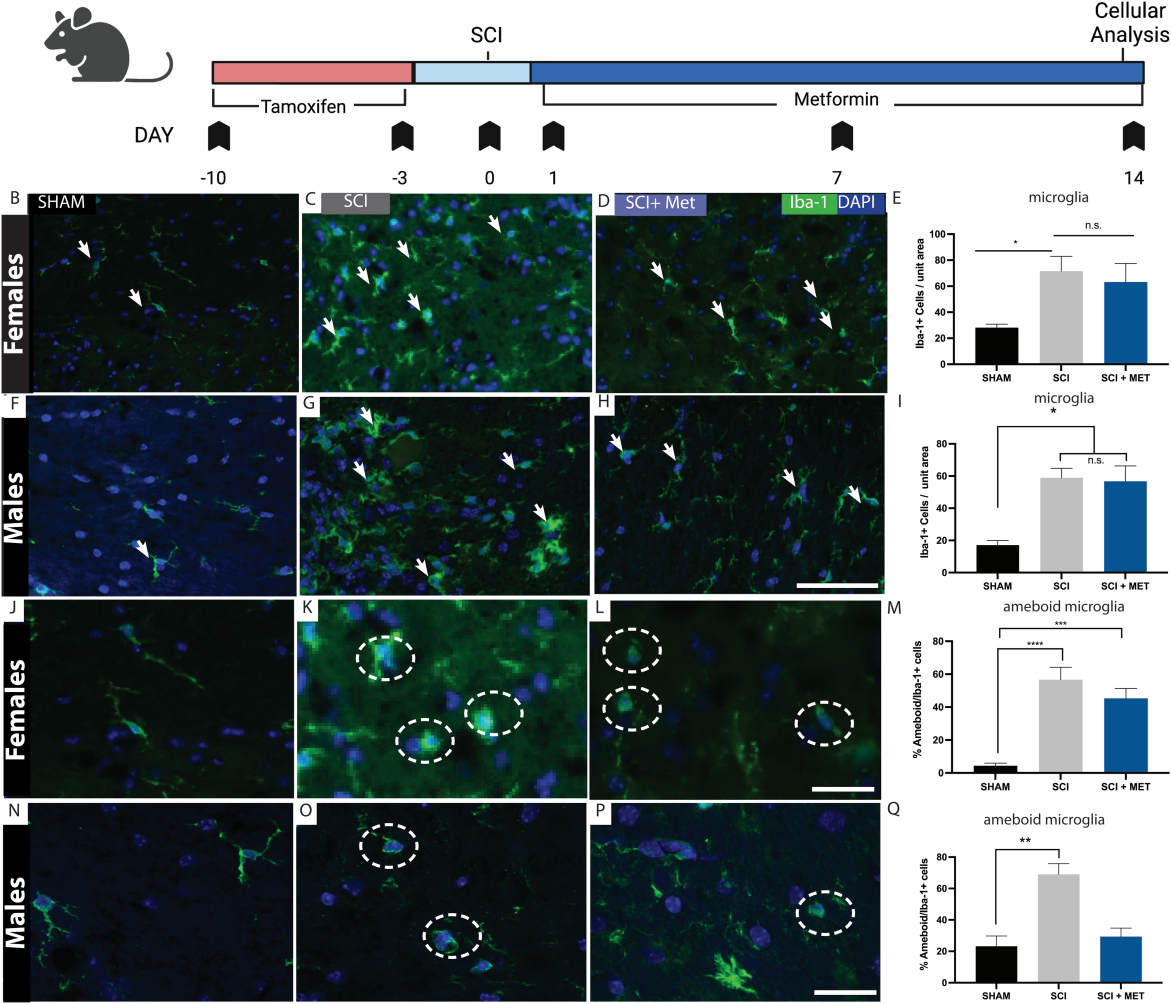
Metformin does not alter the number of microglia following SCI but reduces ameboid microglia in males. (**A**) Experimental timeline. (**B-D**) Iba-1+ microglia were identified (arrows) in (**B)** Sham, **(C)** SCI and **(D)** SCI + Met female mice. **(E)** There is a significant increase in the number of microglia following SCI in females, which was not attenuated in the presence of metformin. **(F-G)** Iba-1+ microglia were identified (arrows) in **(F)** Sham, **(G)** SCI, and **(H)** SCI + Met male mice. **(I)** A significant increase in the number of Iba1+ microglia was observed following SCI in males, which was not attenuated in the presence of metformin. **(J-L)** Iba1+ ameboid microglia (hatched circles) in **(J)** Sham, **(K)** SCI and **(L)** SCI + Met female mice. **(M)** In females, SCI significantly increased the percentage of ameboid Iba1+ cells. Metformin treatment (SCI + Met group) did not reduce the relative percentage of ameboid microglia. **(N-P)** Iba1+ ameboid microglia (hatched circle) were assessed in **(N)** Sham**, (O)** SCI, and **(P)** SCI + Met male mice. **(Q)** In males, metformin treatment (SCI + Met) resulted in a significant reduction in the percentage of ameboid microglia compared to SCI. Scale bar in H, O = 50 μm. Data are presented as mean ± SEM; *n* = 3-7 mice/group. *P < .05, ***P < .01, ****P < .001. Unit area = 450 μm^2^.

Microglia activation occurs following injury to the CNS. Activation includes increased numbers of microglia and morphological changes from ramified to amoeboid shaped cells.^31,54,55^ Studies have shown that metformin reduces microglia activation in the brain following injury.^30,36,38^ Accordingly, we sought to determine whether metformin was modulating the microglia response following SCI and if the effects were sex-dependent. We first quantified the numbers of Iba-1+ microglia on PID14 in Sham, SCI, and SCI + Met groups (Fig. 5A). In both females and males, the numbers of Iba-1+ microglia was significantly increased following SCI (females, Sham = 28.13 ± 2.63, SCI = 71.54 ± 11.43 Iba1+ cells/unit area, *P* = .024; males, Sham = 17.07 ± 2.90, SCI = 58.87 ± 5.95 Iba1+ cells/unit area, *P* = .045) and metformin treatment did not reduce the numbers of Iba1+ microglia compared to SCI alone in either sex (females, SCI + Met = 63.21 ± 14.27 Iba1+ cells/unit area, *P* = .851 compared to SCI; males, SCI + Met = 56.70 ± 9.58 Iba-1+ cells/area, *P* = .036 compared to SCI) (Fig. 5F–5I).

As predicted, when we assessed microglia morphology, we found that SCI alone leads to an increase in the percentage of ameboid microglia in both sexes (females, Sham = 4.39 ± 1.55 vs. SCI = 56.64 ± 7.62% ameboid Iba-1+ cells, *P* < .0001; males, Sham = 23.24 ± 6.58 vs. SCI = 69.94 6.84% ameboid Iba-1+ cells, *P* = .003) (Fig. 5J–5Q). Interestingly, metformin treatment reduced the percent ameboid microglia back to Sham values in males (Sham = 23.24 ± 6.58%, SCI+Met = 29.34 ± 5.44% ameboid Iba-1+ cells, *P* = .798) (Fig. 5Q) but not females. Taken together, these findings reveal that metformin treatment has sex-specific effects on microglia activation, reducing the relative percentage of ameboid microglia in males, but not females.

## Discussion

SCI is a life-altering condition that significantly impacts both patients and their caregivers. The incidence of SCI is greatest in adults and more prevalent in males compared to females (2:1 ratio). Advancements in the medical management of SCI have improved, however, limited progress has been seen in treatment options to improve outcomes. Here, we provide compelling new data highlighting the potential of the FDA-approved type II diabetes medication, metformin, to improve functional outcomes following SCI in both males and females. Our results show that it also has potent effects on neural precursor cells (NSPCs and OPCs) in the spinal cord and also demonstrates that the timing of administration has a significant impact on the functional outcomes whereby delaying metformin treatment to the subacute phase is not efficacious at improving behavioral outcomes.

It is well documented in the literature that metformin administration can alter NSPCs in the brain,^27,29,32^ Considering recent work highlighting the sex-dependent effects of metformin in adult brains,^29^ we considered sex as a variable for our spinal cord studies. Similar to what is reported in the brain, we only observed a significant expansion in the NSPC pool in vitro in females. The differentiation profile of neurosphere-derived cells from the spinal cord was also sex-dependent in the presence of metformin, with males but not females, showing increased neurogenesis. The distinction between the expansion of the neural stem cell pool and differentiation is consistent with previous work showing that metformin acts through 2 separate pathways, the TAp73 and aPKC-CBP pathways, respectively.^33^ Interestingly, across both sexes we observed an increase in oligodendrogenesis similar to what has been reported following metformin treatment of OPCs derived from the brain.^28,56^ To our knowledge this is the first report of metformin expanding the NSPC population in the spinal cord in a sex-dependent manner.

Functional outcomes were improved with metformin treatment when administration began in the acute phase post-SCI. However, delayed administration beginning on PID7 did not promote improved behavioral outcomes. This is different from what was seen following neonatal brain injury where improved functional outcomes were seen following both acute and subacute metformin treatment.^27,36^ It is well established that the NSPC niche is regionally distinct between sexes and through aging which could account for the differences observed.^29,57-59^

In both females and males, we observed a significant increase in OPCs migrating to the lesion site, as well as maturation of OPC-derived cells following SCI in animals that received metformin. Considering we only observed a significant increase in NSPC-derived cells in the injury site in females, yet observed functional recovery across both sexes, we propose that enhancing OPC migration and differentiation rapidly following SCI is critical for promoting improved functional outcomes. This is consistent with the knowledge that demyelination following SCI is a major contributor to the impaired functional outcomes^60,61^ and studies that show that myelinating cells (OPC- or NSPC-derived oligodendrocytes) transplanted into the injured spinal cord promote neural repair in rodent models^62-64^ and in clinical trials.^5,65-67^ Considering previous studies highlighting the effect of metformin on enhancing autophagy,^39-41^ reducing apoptosis^39,68^ and improving the clearance of myelin debris following SCI,^43^ we hypothesize that the pleiotropic effects of this drug on the cellular microenvironment could be providing a more permissive environment for OPCs to proliferate and mature. Promoting neural repair through endogenous precursor cell activation provides a unique opportunity to reduce extrinsic challenges of transplantation including immune rejection and tumorigenicity.

Modulating the inflammatory response has been shown to impact injury repair in the CNS. Herein, we observed changes in microglia activation following SCI and metformin treatment. Indeed, it is well described that altering the microglia/macrophage response can alter functional outcomes.^69-72^ In vitro and in vivo metformin treatment increases AMPK phosphorylation in microglia, which inhibits the expression of pro-inflammatory cytokines without affecting cell viability or expression of ­anti-inflammatory cytokines, skewing activated microglia toward a more anti-inflammatory phenotype.^30,73^ Herein we observed that metformin administration reduced microglia activation similar to what has been shown in the brain^30,36^ and spinal cord.^38,43^ Most interesting, we identified a sex-dependent effect of metformin on microglia activation with reduced ameboid microglia in male mice, but not in female mice, consistent with previous results in a different model of spinal cord injury.^38^ While differences in microglia across the sexes have remained relatively unexplored, recent literature suggests that differences in male and female microglia are present across the life span and can differ in their activation state, functions, and responses following injury.^74-76^ Further, the reduced microglia response following injury could lead to the neuroprotection of NSPCs and OPCs thereby increasing the size of the precursor pools that can contribute to neurorepair.^77,78^

## Conclusion

We have shown that metformin has pleiotropic, sex-dependent effects on the spinal cord. Metformin administration modulates microglia activation and resident precursor cells in terms of their expansion and differentiation and supports improved functional outcomes following SCI. Metformin has a strong safety profile and is readily translatable to the clinic. The reported findings provide a foundation to explore the efficacy of a metformin-mediated endogenous repair strategy to treat SCI with the promise of improved patient outcomes.

## Supplementary Material

szad030_suppl_Supplementary_Material

## Data Availability

All data available are in the main text and supplementary materials. The data that support the findings of this study are available from the corresponding author, C.M.M. upon reasonable request.
