# Supplementary material for: Metformin Improves Functional Outcomes, Activates Neural Precursor Cells, and Modulates Microglia in a Sex-Dependent Manner After Spinal Cord Injury
Source: Stem Cells Transl Med. 2023 May 20;12(6):415–28. doi: 10.1093/stcltm/szad030 (PMC10267579; doi:10.1093/stcltm/szad030)
Supplement: szad030_suppl_Supplementary_Material [file szad030_suppl_supplementary_material.pdf]

| | Sham | | SCI | | SCI + Met | |
|---|---|---|---|---|---|---|
| **Analysis** | *Males* | *Females* | *Males* | *Females* | *Males* | *Females* |
| *Immediate Behaviour (Ladder)* | n=12 | n=13 | n=8 | n=6 | n=6 | n=13 |
| *Immediate Behaviour (Catwalk)* | n=7 | n=6 | n=11 | n=7 | n=8 | n=9 |
| *NSPC lineage tracking* | n=3 | n=3 | n=6 | n=3 | n=5 | n=3 |
| *OPC lineage tracking* | n=4 | n=6 | n=3 | n=4 | n=3 | n=4 |
| *Microglia analyses* | n=3 | n=6 | n=4 | n=5 | n=7 | n=6 |
| *Delayed Behaviour (Ladder)* | n=7 | n=11 | n=9 | n=6 | n=10 | n=9 |
| *Delayed Behaviour (Catwalk)* | n=7 | n=11 | n=9 | n=6 | n=10 | n=9 |
| | **Vehicle** | | | **Met** | | |
| **Analysis** | *Males* | *Females* | | *Males* | *Females* | |
| *In vitro analysis* | n=9 | n=9 | | n=9 | n=9 | |

**Supplemental Table 1:** Summary of number of mice for all analyses

Supplemental Figure 1

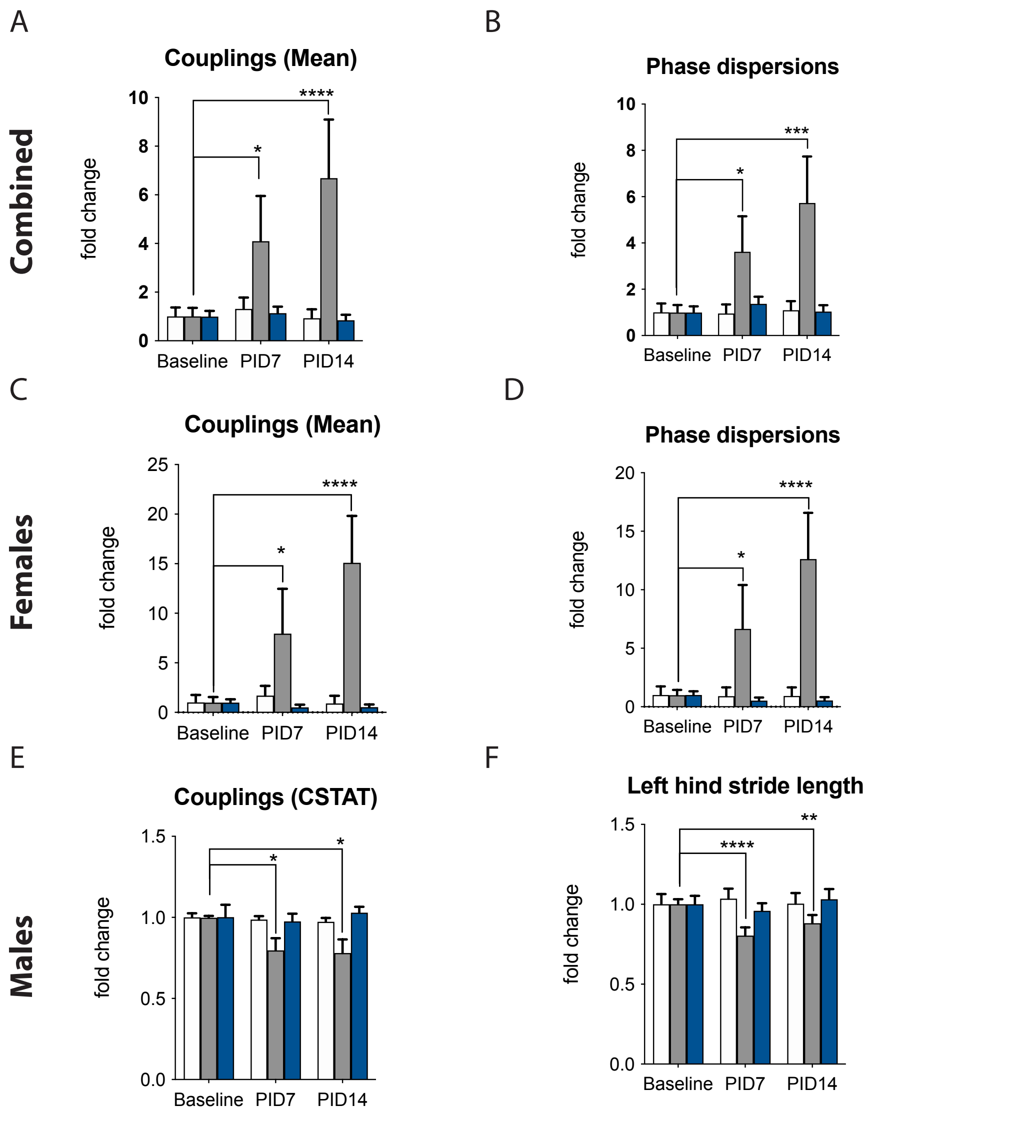

Supplemental Figure 2

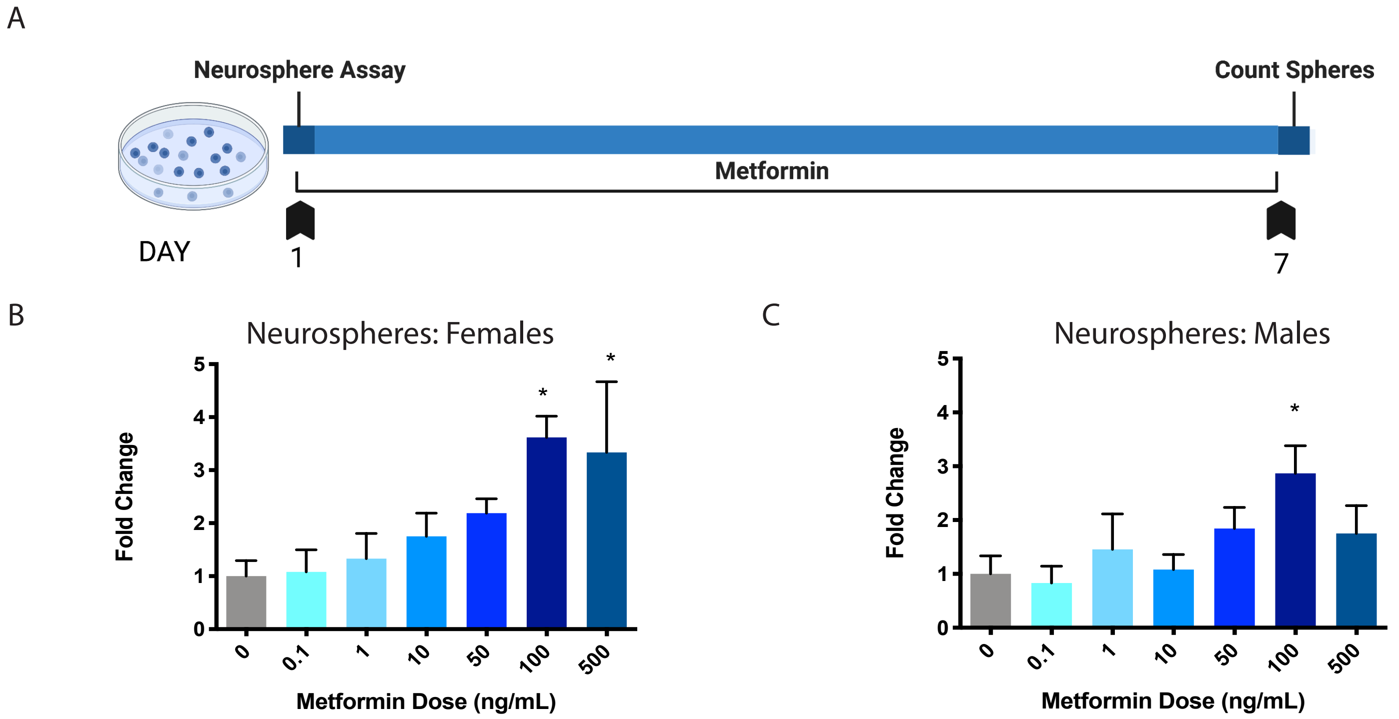

Supplemental Figure 3

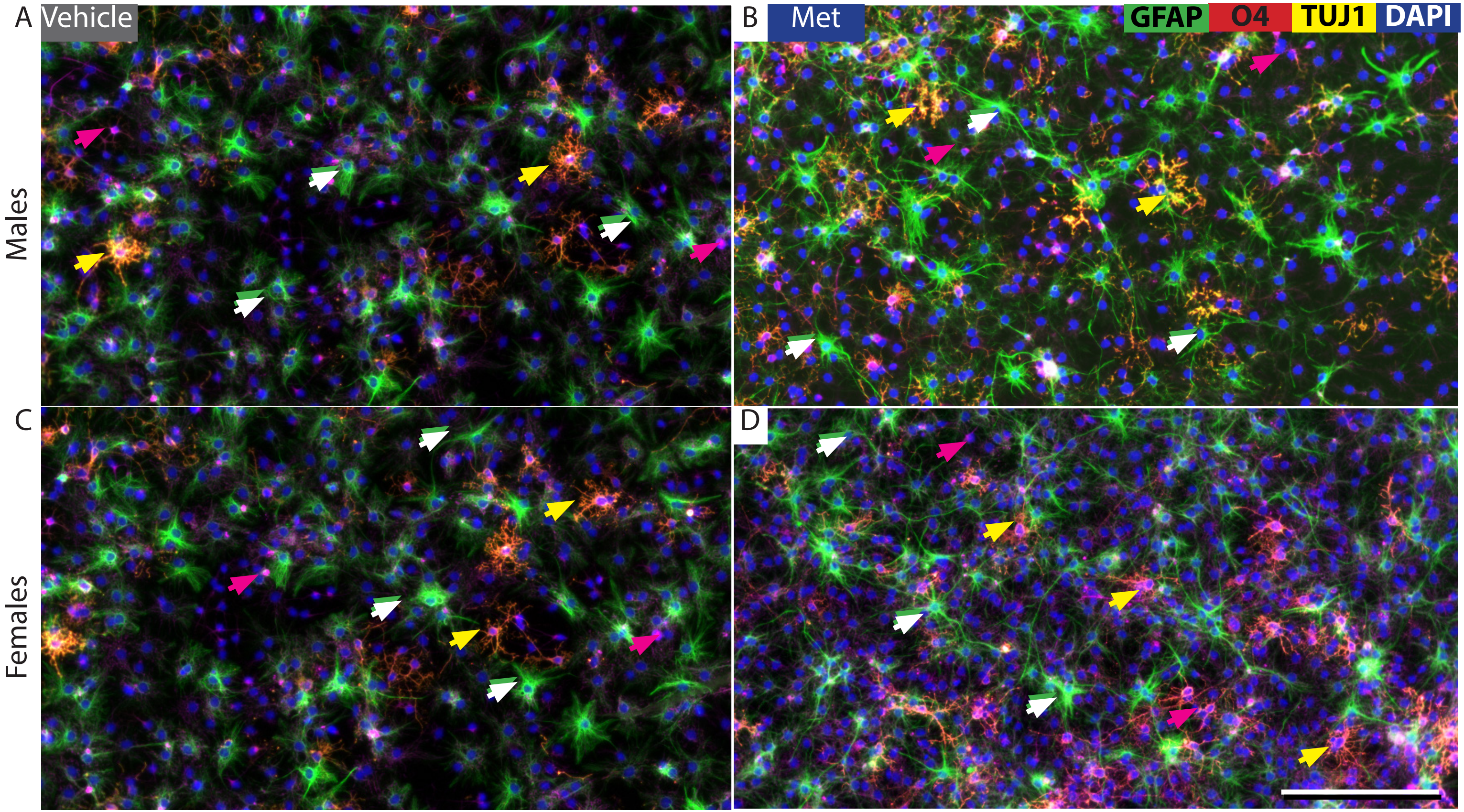

Supplemental Figure 4

A **Females:** EdU+/TdTomato+ cells in dorsal cord

B **Males:** EdU+/TdTomato+ cells in dorsal cord

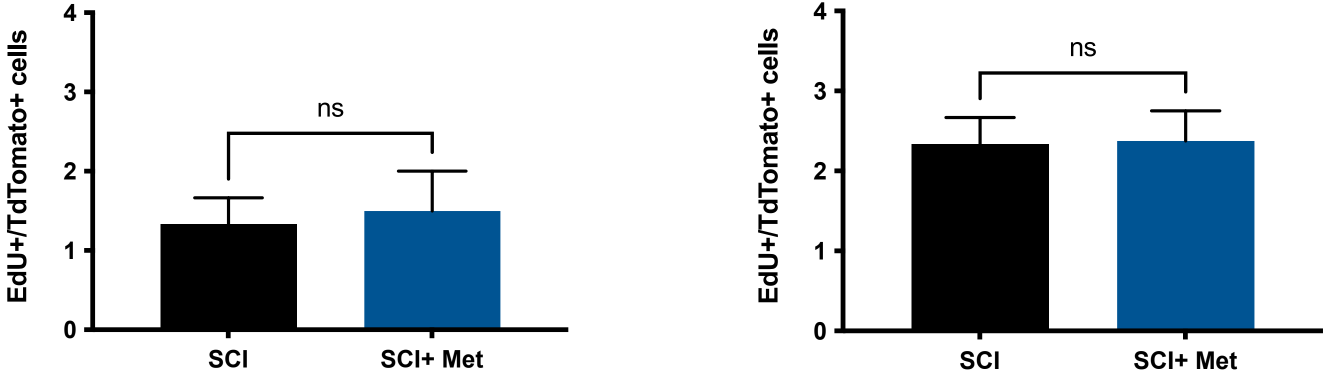

**Supplemental Fig 1: Immediate metformin improves gait following SCI.** Deficits in gait were observed in SCI mice in our acute studies. Mice that received metformin treatment (SCI+Met) were not impaired in any of these gait parameters at PID7 or PID14. **(A,B)** In our combined groups, mean couplings **(A)** and phase dispersion **(B)** were significantly impaired in SCI, but not SCI+Met mice. **(C, D)** Females had significant deficits in mean couplings **(C)** and phase dispersion **(D)** in SCI, but not SCI+Met mice**. (E,F)** Males demonstrated significant deficits in couplings (CSTAT) **(E)** and left hind stride length **(F)** in SCI, but not SCI+Met mice**.** Data are presented as mean ± SEM; n=18-25/group including males and females; in data split by sex, n=6-11/group. *$p<0.05$.

**Supplemental Fig. 2: *In vitro* dose-response curves for metformin. (A)** *In vitro* experimental timeline. **(B)** *In vitro* delivery of metformin in females reveals a significant increase in the number of neurospheres at both 100ng/mL and 500ng/mL. **(C)** In males, a significant increase in the number of neurospheres is observed at 100ng/mL metformin. n=9 mice/group *$p<0.05$

**Supplemental Fig. 3: Neurospheres are multi-potent in both males and females in the presence or absence of metformin. (A)** male, vehicle. **(B)** male, metformin. **(C)** female, vehicle. **(D)** female, metformin. Scale bar in D= 200μm, n=12 neurospheres/group from 9 mice.

**Supplemental Fig. 4: Rare Edu+/TdTomato+ cells are observed in the dorsal spinal cord in females and males.** (A,B) There is no significant difference in EdU+/TdTom+ cells in the dorsal (injured) spinal cord in females (A) or males (B) between SCI and SCI+Met treated mice. Data are presented as mean ± SEM; n=3-4 mice/group.
